# Clinical Applications of Targeted Nanomaterials

**DOI:** 10.3390/pharmaceutics17030379

**Published:** 2025-03-17

**Authors:** Ankesh Kumar, SK Shahvej, Pankaj Yadav, Unnati Modi, Amit K. Yadav, Raghu Solanki, Dhiraj Bhatia

**Affiliations:** 1 Department of Biological Sciences and Engineering, Indian Institute of Technology Gandhinagar, Palaj, Gandhinagar 382355, Gujarat, India; 2 Amrita School of Biotechnology, Amrita Vishwa Vidyapeetham, Kollam 690525, Kerala, India

**Keywords:** nanomaterials, clinical applications, drug delivery

## Abstract

Targeted nanomaterials are at the forefront of advancements in nanomedicine due to their unique and versatile properties. These include nanoscale size, shape, surface chemistry, mechanical flexibility, fluorescence, optical behavior, magnetic and electronic characteristics, as well as biocompatibility and biodegradability. These attributes enable their application across diverse fields, including drug delivery. This review explores the fundamental characteristics of nanomaterials and emphasizes their importance in clinical applications. It further delves into methodologies for nanoparticle programming alongside discussions on clinical trials and case studies. We discussed some of the promising nanomaterials, such as polymeric nanoparticles, carbon-based nanoparticles, and metallic nanoparticles, and their role in biomedical applications. This review underscores significant advancements in translating nanomaterials into clinical applications and highlights the potential of these innovative approaches in revolutionizing the medical field.

## 1. Introduction

**Figure 1:**
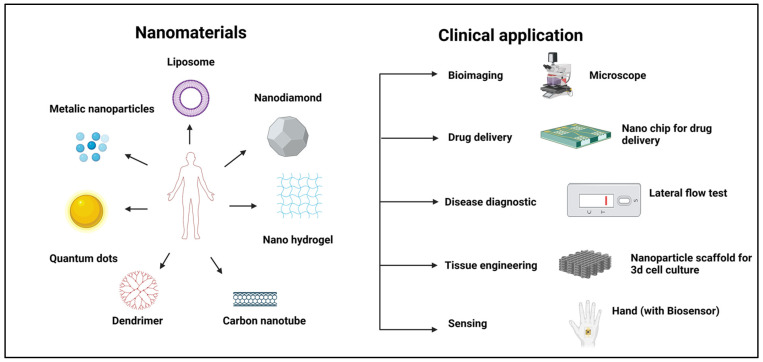
Different types of nanomaterials and their clinical applications.

In recent years, nanotechnology has gained significant attention and has positively influenced preclinical medical research and advancements in the emerging field of nanomedicine. Nanomaterials are now used to engineer nano-systems that help in targeted drug delivery and disease treatment. Nanomaterials offer several benefits across various fields including bioimaging, drug delivery [1], regenerative medicine [2], cosmetics [3], electrochemical DNA detection [4], polishing [5], energy storage [6], sunscreen protection [7], and sensing [8] (Figure 1).

Nanoparticles have unique and advantageous properties, such as chemical, physical, and biological properties [9]. Various essential nanomaterial platforms have been developed for biomedical applications, including in drug delivery and cancer therapy [10]. Nanomaterials can deliver a wide range of therapeutic and diagnostic payloads such as small molecule drugs, nucleic acids, proteins, and imaging agents [11]. These nanoparticles can be specifically programmed to enhance the safety and efficacy of drug delivery [12]. Engineered nanoparticles hold great promise for disease diagnosis and treatment; however, they must overcome various biological barriers. Advances in nanoparticle engineering and understanding their physicochemical properties have led to new therapeutic opportunities [13]. Pharmaceutical nanocarriers like liposomes and micelles exhibit enhanced stability, targeted delivery, improved penetration, and imaging contrast properties. The development of multifunctional nanocarriers has the potential to significantly enhance both therapeutic and diagnostic efficacy [14]. Potential nanomaterials include quantum dots, dendrimers, polymers, carbon nanotubes, organic nanoparticles, metallic nanoparticles, liposomes, nanogels, and peptide-based nanoparticles.

Nanoparticles (NPs) smaller than 5 nm are easily eliminated from the systemic circulation, while those larger than 10 nm tend to persist in the bloodstream. Expanding nanomedicine from small-scale to large-scale production while maintaining NPs’ stability presents significant challenges [15]. Polyethylene glycol (PEG) is a widely used polymer that forms hydrophilic chains to resist protein absorption; however, subsequent opsonization and macrophage-mediated clearance can still occur. Strategies like liposomes modified with albumin on their surface have demonstrated prolonged circulation times compared to both unmodified and PEGylated versions [16]. Cationic polymers encapsulate siRNA, increasing cellular uptake despite their toxicity, thus overcoming barriers to targeted drug delivery [17]. Liposomes with stimuli-responsive characteristics like light, pH, temperature, enzymatic reaction, ultrasound, or radiation are effective for multimodal nanoscale functions [18]. Cationic poly(amidoamine) (PAMAM) dendrimers interact with negatively charged molecules, showing toxicity, but zwitterionic dendrimers with phosphorylcholine-modified surfaces have been shown to reduce cytotoxicity [19].

There are various nanomaterials that have been used for biomedical applications, with some progressing to clinical trials and receiving food and drug administration (FDA) approval. For example, Abraxane is an albumin-bound paclitaxel and was approved in 2007 for metastatic breast cancer [20]. Another example is Doxil, a PEGylated liposomal formulation of doxorubicin, which is now widely used for various cancers [21]. Additionally, Patisiran received FDA approval for the treatment of hereditary transthyretin amyloidosis [22], while nab-paclitaxel in combination with gemcitabine has been approved for pancreatic cancer therapy [23]. CALAA-01 is the first targeted nanoparticle-based system for siRNA delivery that successfully completed preclinical trials and entered human clinical trials [24]. Designed for systemic nucleic acid administration in cancer therapy, it uses cyclodextrin-containing polymers (CDPs) to form nanoparticles of approximately 70 nm [24].

In this review, we discuss various clinically approved nanomedicines and those currently being used in clinical trials. We begin with the historical background and fundamental characteristics of nanoparticles, followed by the key requirements for programming nanoparticles, various types of nanoparticles, clinical trials and case studies, and their clinical applications. Additionally, we address the challenges associated with nanoparticle programming. This review provides an in-depth examination of the evolution of nanotechnology, the diverse applications of nanoparticles in biomedicines, and the technical challenges in optimizing their functionality for therapeutic use.

## 2. Historical Background of Programmable Nanomaterials

**Figure 2:**
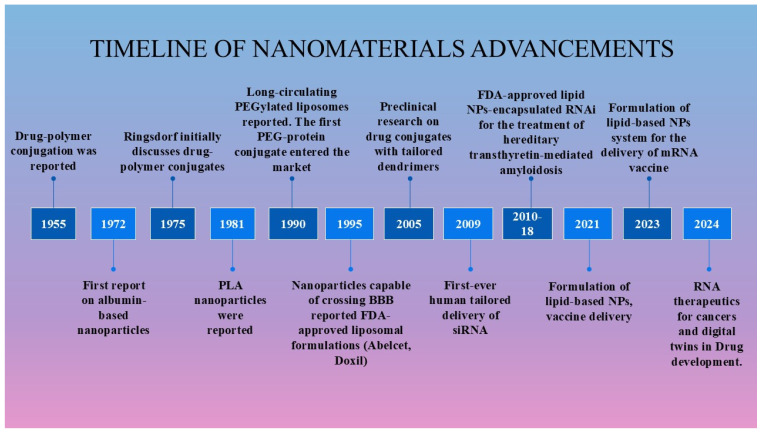
Historical progress in nanoparticle-based therapies.

In 1959, physicist Richard Feynman first introduced the concept of nanotechnology in his lecture, “There’s Plenty of Room at the Bottom” [25,26]. This visionary idea laid the foundation for significant advancements in the field of nanotechnology. Nanotechnology involves the study and engineering of nanoscale materials, generally ranging from 1 to 100 nm, and serves as an interdisciplinary field consisting of physics, chemistry, biology, engineering, and materials science. The evolution of nanomedicine from ancient applications to its critical role in modern healthcare demonstrates an integration of history, science, and technology. This path has been greatly affected by the advancement of molecular biology, which has highlighted essential macromolecules such as DNA, RNA, and peptides. Currently, over 500 nanoformulations are undergoing clinical studies with the goal of introducing innovative clinical solutions to the market [27]. Microfluidics, materials, robotics, photonics, biotechnologies, and smart systems are some of the enabling technologies that are being developed alongside or independently of improved nanoformulations. The evolution of nanomaterials exemplifies the convergence of human ingenuity and scientific progress, leading to the FDA approval of diverse nanoformulations including polymeric nanoparticles, liposomes, lipid-based nanoparticles, dendrimers, and polymersomes for the treatment of various diseases (Figure 2).

In 1955, von Horst Jatzkewitz reported the first synthesis of the drug Mescaline conjugated with poly(vinyl)pyrrolidine via a small peptide spacer between the polymer and the drug [28]. The first lipid-based nanotechnology drug delivery system, later known as a liposome, was developed in the 1960s [29]. In the 1970s, Ringsdorf introduced a targeted polymer drug carrier idea, which eventually entered clinical trials [30]. The approach, known as Ringsdorf’s model, had a polymeric backbone, a linker for drug conjugation to the backbone, a drug, and a targeting group [30,31]. The concept of controlled drug delivery was introduced in 1976, marking a significant advancement that redirected the focus of researchers toward precision medicine. In the mid-1970s, soluble polymers were first proposed as targetable drug carriers [32]. While early polymer–drug conjugates remained in the developmental stage, some conjugates like monomethoxy polyethylene glycol asparaginase (mPEG–asparaginase) and styrene maleic acid neocarzinostatin (SMANCS) have progressed to clinical evaluation. The polymer–drug poly(styrene co-maleic acid) was conjugated with the cytotoxic drug neocarzinostatin [28,33].

The first FDA approval of liposomes occurred in the 1990s, despite three decades of research on their efficacy as a drug delivery system for various therapeutic agents. Doxil^®^, a PEGylated liposomal formulation of doxorubicin, marked this milestone, and has been approved for the treatment of ovarian cancer, metastatic breast cancer, and multiple myeloma [34]. Zoladex^®^ is another example of a biodegradable and injectable nano-drug delivery system developed by AstraZeneca [35]. It is used for the palliative treatment of advanced breast and prostate cancer. Its formulation consists of a sterile, biodegradable polymer matrix that encapsulates goserelin acetate, which is a luteinizing hormone releasing hormone (LHRH) agonist. In the 1990s, significant milestones were achieved in the advancement of nanoparticle-based drug delivery systems. In 1999, the first dendrimer–drug conjugate designed for tumor treatment entered clinical trials [36]. Then, synthetic polymer–anticancer drug conjugates also entered clinical trials [37]. SP1049C is an innovative doxorubicin formulation of two nonanoic pluronic block copolymers (pluronic L61 and pluronic F127) and doxorubicin, which has demonstrated greater efficacy than doxorubicin in preclinical studies [38].

Abraxane (nab-paclitaxel), also known as paclitaxe–albumin nanoparticles, has an estimated diameter of 130 nm, in 2005, was the first FDA-approved nanotechnology-based chemotherapy, demonstrating significant efficacy in the treatment of metastatic breast cancer [39,40]. Its market approval represents a landmark achievement, not only for albumin-based drug delivery technology but also for the broader field of nanomedicine [41,42]. Researchers are also actively developing siRNA-based therapies for various diseases, driven by the success of several FDA-approved nanomaterials. The first targeted delivery of siRNA in humans was achieved in 2008 during a Phase I clinical trial using an investigational treatment with CALAA-01 [24]. This targeted nanoparticle was administrated intravenously for the treatment of solid tumors. In 2018, the FDA approved Patisiran (Onpattro), the first siRNA-based drug for the treatment of polyneuropathy in patients with hereditary transthyretin (TTR)-mediated amyloidosis [43]. In 2019, givosiran (Givlaari) received FDA approval for the treatment of acute hepatic porphyria. This was followed by the approval of lumasiran, the third siRNA-based therapy, for managing primary hyperoxaluria [43]. For the treatment of familial hypercholesterolemia (FH), the FDA approved inclisiran in 2022 as an adjuvant to a better diet and the administration of statins [44]. Additionally, multiple siRNA-based treatments are currently undergoing clinical trials. Recently, the FDA granted Emergency Use Authorization (EUA) to two mRNA-based COVID-19 vaccines: Spikevax (Moderna) and Comirnaty (Pfizer-BioNTech) [45,46]. These lipid nanoparticle-based formulations offer several advantages, including enhanced cellular uptake, the protection of mRNA from degradation, and efficient endosomal escape.

## 3. Characteristics of Nanomaterials

**Figure 3:**
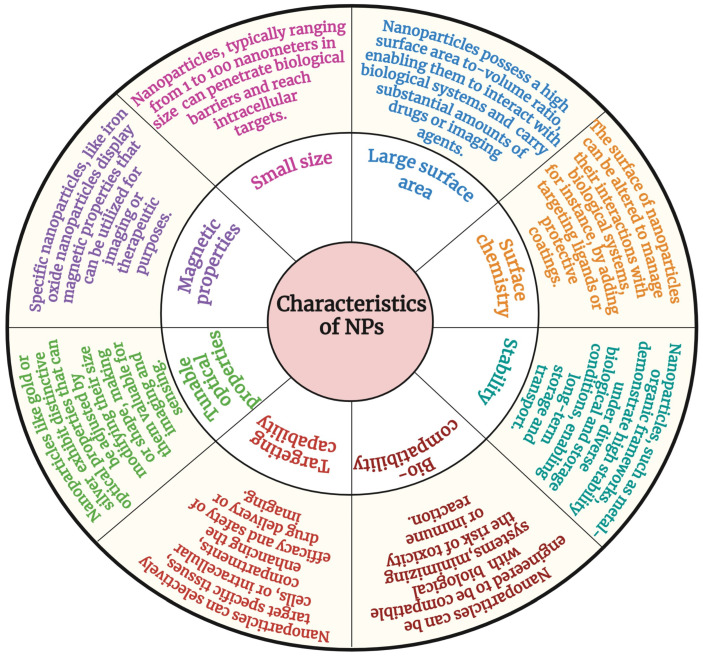
Characteristics of nanomaterials.

Nanoparticles have unique physicochemical properties that make them applicable to various clinical applications (Figure 3). The most essential properties of nanoparticles are their nanoscale size (1–100 nm), which allows them to circulate within the body without affecting blood circulation. The size and shape of nanomaterials play a crucial role in cellular uptake [47]. Particles of less than 5 nm are easily eliminated from the systemic circulation via renal clearance, whereas nanoparticles ranging from 10 nm to 100 nm accumulate in the spleen, liver, and bone marrow [48,49]. The biodistribution of nanoparticles within the 10–100 nm range varies depending on cell type, with cellular uptake being influenced by factors such as surface charge and ligand functionalization [50,51]. Additionally, nanomaterial accumulation in specific cells is detected by a specific type of protein that absorbs in vivo [52,53]. This mechanism is known as opsonisation, which occurs upon interaction with plasma protein and is significantly influenced by surface modifications of nanomaterials [54].

Nanomaterials exhibit distinct characteristics that differentiate them from their bulk counterparts. As materials transition from bulk to the nanoscale, certain physical properties such as color and phase undergo significant changes [55]. For example, gold, which appears yellow in its bulk form, transforms into a purple or red solution at the nanoscale due to surface plasmon resonance effects. Studies suggest that the enhanced mechanical, optical, and electrical properties observed at the nanoscale can be attributed to factors such as reduced crystallographic defects and improved crystal lattice perfection [56]. These unique nanoscale properties have profound implications for applications in nanomedicine, photonics, electronics, and materials science.

Metallic NPs have fascinated researchers for decades, and are now broadly utilized in biomedical engineering [57,58,59]. These nanoparticles possess unique properties, such as distinctive optical and electronic characteristics. This is due to the free electrons in the metal nanoparticles, which are collectively excited in response to incident light [60]. Specifically, metallic and metal oxide nanoparticles possess magnetic properties, making them suitable for various applications, including imaging, cell separation, targeting, and drug delivery. Nanomedicine formation is easy on a small scale, but expanding nanomedicine from small-scale to large-scale production while maintaining stability presents significant challenges [15].

Recent approaches to mitigating the negative effects of opsonisation have focused on slowing the process by making the particle surface more hydrophilic or by balancing the surface charge of the nanoparticles. The most common process is adsorbing or grafting a hydrophilic polymeric coat, for example, PEG, on the nanoparticle surface [61,62,63]. PEG is well-known for its high hydration capacity, biocompatibility, amphiphilicity, and ability to reduce steric interactions, making it widely used in biomedical applications. In one particular study, liposomes with albumin covalently attached to their surface ex vivo demonstrated longer flow times than their unmodified or PEGylated versions [16]. When exposed to plasma, the researchers attributed this extended flow to decreased opsonin binding.

Cell culture and body fluids are mainly made from salts, mineral ions, and biomolecules. In their study, Allouni et al. found that the stability of nanomaterials is determined by many factors in culture media, for example, the concentration of nanoparticles, protein abundance, or ionic strength; these salt and mineral ions are intermingled with a nanomaterial for their charge compatibility, frequently leading to the precipitation and agglomeration of the nanomaterial [64].

## 4. Surface Coating and Functionalization Strategies to Enhance Nanomaterial Properties

Nanomedicine involves the manipulation of human biological systems at the molecular level using nanoscale or nanostructured materials [65,66]. These materials interact effectively with biological systems, providing promising solutions to various challenging health problems through nano-diagnostics and nanotherapeutics. Various nanoparticles have been created with modified functional surfaces and bioactive cores. This design provides several beneficial therapeutic and diagnostic properties, including improved permeation and retention in the circulatory system, targeted drug delivery, highly efficient gene transfection, and advanced bioimaging capabilities [67]. Surface coatings were initially developed for industrial applications to prevent corrosion and enhance mechanical resistance. With advancements in nanoscience, polymers and nanocomposite-based coatings have been engineered for diverse applications, including biomedical uses such as antibacterial surfaces [68].

Several studies have investigated the influence of physicochemical parameters on the absorption, transport, and fate of programmable nanomaterials. Key physicochemical properties that govern nanomaterials include: (1) surface chemistry, (2) physical properties such as size, shape, and surface area, (3) surface modifications under biological conditions, (4) dispersion, aggregation, and agglomeration behavior, and (5) stability under physiological conditions. These properties collectively influence nanomaterial biodistribution, cellular uptake, and potential toxicity, making their precise characterization essential for biomedical applications [69].

Stimuli-responsive nanocarriers, capable of altering their characteristics in response to internal or external triggers, have revolutionized biomedical applications [70]. These smartly designed nanocarriers allow for exact drug delivery at the target spot, increasing therapeutic efficacy while limiting harmful effects [71]. Different stimuli-responsive nanocarriers are developed based on intrinsic or extrinsic responsive stimuli. Amphiphilic compounds, including natural lipids, surfactants, and responsive polymers, play a key role in nanocarrier design. Some are engineered with cationic modifications to facilitate loading anionic biomolecules like nucleic acids, enhancing their stability and delivery efficiency [72].

Intrinsic stimuli-responsive nanocarriers are designed to respond to the tumor microenvironment due to pH changes, enzymes, oxidative stress, and glutathione, and these stimuli trigger drug release. Glutathione (GSH), a redox agent, is significantly higher in cancer cells (2–10 mM) than in normal tissues (2–10 μM). It can influence drug delivery and release in a controlled manner [73,74]. Glutathione converts disulfide bonds into free thiol groups, whereas diselenide bonds are even easier to break under redox circumstances due to their lower bond energy [75,76,77]. Choi et al. designed a graphene oxide-based nanocarrier [78], where a photosensitizer is attached via a redox-sensitive disulfide bond. Under normal conditions, even with light exposure, the photosensitizer remains dormant—neither fluorescent nor toxic. However, when the nanocarrier reaches tumor tissues rich in glutathione, the bond is cleaved, reactivating the photosensitizer to emit fluorescence and generate singlet oxygen. This GSH-triggered system holds promise for photodynamic therapy and fluorescence imaging. Hypoxia-sensitive nanocarriers are designed to utilize this condition. One strategy is like nanocarriers, which use a light-sensitive polymer paired with hypoxia-sensitive 2-nitroimidazole. When exposed to laser light, the polymers produce ROS and create a locally low-oxygen (hypoxic) environment. This change causes the 2-nitroimidazole attached to the polymers to switch from being water-repellent (hydrophobic) to water-attracting (hydrophilic), triggering doxorubicin release for improved anticancer effects [79]. Also, hypoxia-sensitive nanocarriers improve both tumor imaging and therapy. For example, nanoparticles loaded with oxygen-sensitive iridium (III) complexes—such as Ir-PVP and PCL-PVP—enable optical imaging of tumors and metastases [80].

External stimuli—such as heat, magnetic or electric fields, ultrasound, and light—play a crucial role in guiding nanocarriers within the body. These external stimuli help to concentrate nanocarriers at target sites, enable on-demand drug release, enhance delivery into cells, and even activate imaging and therapeutic functions [81]. Thermo-responsive nanocarriers are built with temperature-sensitive materials that let them hold onto their drug load at a normal body temperature. When they reach warmer areas—like sites of inflammation, injury, infection, or cancer—the heat triggers them to release the drug exactly where it is needed [82]. Poly(N-isopropyl acrylamide) (PNIPAM) is widely used in temperature-responsive nanocarriers due to its unique thermal transition properties [83,84]. One study showed that siRNA–SS–PNIPAM conjugates can self-assemble into structures called siRNAsomes when heated above 32 °C, which is the LCST for PNIPAM [85]. In another example, nanocarriers featuring PNIPAM on their surface formed micellar aggregates at temperatures above the LCST, but disassembled when the temperature dropped below this threshold and helped in site-specific drug release [86].

Circulating nanoparticles can aggregate due to van der Waals and hydrophobic interactions, leading to reduced circulation time and potential clearance by the MPs. In high-ionic-strength environments such as blood, electrostatic interactions between nanoparticles and counterions neutralize surface charges, promoting nanoparticle aggregation and potential entrapment in pulmonary capillaries [87]. The stealth effect plays a crucial role in enabling nanomaterials for drug delivery applications, as it enhances pharmacokinetics by improving blood circulation, biodistribution, and tissue targeting [88]. PEGylation is particularly effective in sterically hindering interactions between nanoparticles and the surrounding components due to their hydrophilic nature, which helps form a hydrated cloud with a high excluded volume. In the 1970s, Abuchowski and Davis discovered that PEGylation enhances protein circulation and reduces immunogenicity [89]. Similarly, Illum and Davis demonstrated that steric repulsion could extend blood circulation using polystyrene latex models [90]. In another study, matrix metalloproteinase (MMP)-mediated dePEGylation of polymersomes was designed by Li Junjie et al., which enhances the tissue and cellular targeting/retention while increasing bioavailability [91]. Additionally, this process enables the differential release of marimastat and colchicine, allowing them to act on their respective extracellular (MMPs) and intracellular (microtubules) targets.

The surface functionalization of nanomaterials involves the use of covalent and non-covalent interactions, including hydrogen bonding, electrostatic forces, and van der Waals interactions to integrate various organic and inorganic compounds at the nanoscale. Typically, linker molecules facilitate covalent bonding between ligands and nanomaterial surfaces ensure stability and targeting functionalization [92]. The clinical applications of programmed nanomaterials in therapeutics and diagnostics tools often requires functionalization with specific biomolecules (e.g., peptides, ligands) or chemical groups to facilitate the targeted delivery of drugs, bioactive molecules, or nucleic acids [69]. The affinity and density of these surface biomolecules or chemical groups play a crucial role in regulating nanoparticle interactions with membrane receptors. For example, sialic acid receptors are overexpressed on solid tumor cell surfaces and are associated with cancer progression, apoptosis, and resistance to chemotherapy [93]. Phenylboronic acid (PBA) can specifically recognize and bind to sialic acid receptors on cancer cell surfaces, making it a potential tool for targeted cancer therapy. We showed that pullulan nanoparticles functionalized with PBA exhibit significantly enhanced cellular uptake in A431 cells compared to unmodified pullulan [94]. Additionally, PBA facilitates the improved uptake of PAMAM dendrimers [95] and PEG–steric acid biomaterial-based nanomicelles in cancer cells [96]. Folic acid (FA) is another well-known targeting ligand for folic acid receptors (FRs), which are overexpressed on cancer cell surfaces [97]. Conjugating FA to nanoparticles enhances the targeted delivery of anticancer agents and phytochemicals through FR-mediated endocytosis and improves therapeutic efficacy [98,99]. In conclusion, the enhanced permeability ad retention (EPR) effect facilitates passive targeting by allowing nanomaterials to accumulate in tumor tissues while evading the P-glycoprotein-mediated efflux in cancer cells. Additionally, active targeting strategies further enhance the precision of nanomaterials in cancer diagnostics and other medical treatments.

## 5. Different Types of Targeted Nanomaterials

Targeted nanomaterials are engineered for functions and applications. This review explores various types of nanoparticles (NPs) and their biological applications, including polymeric NPs, carbon-based NPs, and metallic NPs.

### 5.1. Polymeric Nanomaterials

Polymeric nanomaterials derived from organic matter exhibit uniform distribution and are widely used in biomedical applications. Biodegradable, nontoxic, and biocompatible polymeric nanoparticles (e.g., albumins and chitosan) are widely used in receptor-mediated drug delivery and therapeutics [100]. Polymeric hybrid nanomaterials have achieved significant interest because of their versatile applications in the biomedical sphere. They are instrumental in modifying biological entities, serving as transporters for hydrophobic drugs, and acting as non-viral vectors for nucleic acid delivery. A common approach involves conjugating polymers like PEG with proteins to enhance pharmacokinetics while preserving the biological function of proteins. However, the potential of self-assembled nanoparticles remains underutilized. Polymers can be broadly classified into degradable and non-degradable categories, with both natural and synthetic variants used to fabricate polymeric vesicles and micelles for drug delivery [101,102,103]. Compared to natural polymers, some specific synthetic polymers have long lifetimes, allowing for enhanced disease targeting. They are also used in long-term drug-release (over several days or weeks) systems. Amphiphilic polymers or graft copolymers are constructed in a liquid medium to form nanoparticles. Polymers, for example, PGA (poly-glycolic acid), PLA (poly-lactic acid), and PLA (poly-lactic acid), and their copolymers are the most broadly studied, due to their favorable biodegradability and biocompatibility.

For the past three decades, polymeric-based nanomaterials have been utilized for formulation development, biocompatibility assessments, and surgical applications, demonstrating their suitability for biomedical use [104]. The drug release rate of these nanomaterials is controlled by altering the mass of the polymer nanoparticles. For copolymers, their nanostructure and composition are used to control drug release [105]. Several anticancer drugs such as tamoxifen and paclitaxel have been successfully encapsulated in polymeric nanoparticles. Engineered polymeric-based nanomicelles (10–100 nm) are highly insoluble in water compared to polymeric vesicles, which enhances their accumulation in tumor tissue and improves permeability and retention time within the tumor [106]. Additionally, specific cationic polymers can bind with nucleic acid, facilitating siRNA transport and cellular transfection. This approach holds promise for developing novel therapeutic strategies for treating severe illnesses, including cancer and inherited metabolic diseases. For example, Mao et al. demonstrated the use of a cationic triblock copolymer in cancer therapy to deliver siRNA targeting the acid ceramidase gene. They illustrated how to create siRNA/biodegradable micellar triblock copolymer complexes that effectively penetrate cancer cells and silence genes [107]. However, while cationic polymers effectively encapsulate siRNA, their associated cytotoxicity poses a challenge for clinical translation [17]. Nevertheless, the positively charged catatonic polymer increases cellular uptake; therefore, this approach overcomes barriers in targeted drug delivery [108].

#### 5.1.1. Clinical Examples

##### Genexol-PM and NK105

Genexol-PM, developed by Samyang Biopharm in South Korea, is a lyophilized polymeric micellar nanocarrier containing the paclitaxel (PTX) drug, and is approved for the treatment of MBC, ovarian cancer, pancreatic cancer, and advanced NSCLC. This nanomedicine allows for the administration of the higher maximum tolerable dose and reduces the risk of allergic or adverse reactions to paclitaxel [109,110,111,112]. NK105 is another nanomedicine, developed by Nippon Kayaku Co., Tokyo, Japan. Its particle size is an 85 nm nanoparticle formulation of paclitaxel (PTX) with a hydrophilic PEG outer shell and a hydrophobic core for efficient drug encapsulation [113,114].

##### Abraxane (Nab-Paclitaxel)

The albumin-bound nanoparticle formulation of paclitaxel (nab-paclitaxel) takes advantage of enhanced albumin delivery to tumors via receptor-mediated transport or transcytosis. Nab-paclitaxel promotes caveolin-1 expression and the development of caveolae by binding to the endothelial cells’ gp60 albumin receptor [115]. Studies suggest that the combination of nab-paclitaxel and gemcitabine is associated with improved survival in pancreatic cancer patients. In a Phase III study, patients with metastatic pancreatic cancer were randomly assigned to receive nab-paclitaxel (125 mg/m^2^) plus gemcitabine (1000 mg/m^2^) or gemcitabine alone. The primary endpoint was overall survival. Among 861 patients, the nab-paclitaxel–gemcitabine group had a median overall survival of 8.5 months compared to 6.7 months in the gemcitabine group (hazard ratio, 0.72; *p* < 0.001). The one-year survival rate was 35% versus 22%, respectively. Progression-free survival was 5.5 months versus 3.7 months (hazard ratio, 0.69; *p* < 0.001). The response rate was 23% in the combination group versus 7% in the monotherapy group (*p* < 0.001). Higher rates of neutropenia, fatigue, and neuropathy were observed with the combination treatment [116].

### 5.2. Lipid-Based Nanomaterials

Lipid-based nanomaterials have been developed to address the challenges associated with the solubility and bioavailability of poorly water-soluble drugs [117]. Lipid-based nanomaterials encompass a diverse range of nanocarriers including liposomes, solid lipid nanoparticles, and lipid nanostructures. Liposomes make up a vesicular carrier system based on phospholipids and are widely used as a potential drug delivery vehicle. Liposomes have several benefits over traditional delivery systems, including the capacity to target specific sites and boost stability, controlled release, and they also have lower associated toxicity. Many researchers have worked to engineer primary or modified liposomes in the last ten years to deliver a range of therapeutic agents efficiently [118]. The fundamental structural elements of liposomes include a phospholipid bilayer, cholesterol, lipoproteins, a hydrophilic core, and targeting ligands like amino acid fragments, antibodies, or proteins for specific cell targeting [119].

The new approaches, for example, surface engineering, have enabled the development of liposomes with tailored surface characteristics, allowing for targeted delivery to cancerous cells. Due to their localized retention, certain liposomes function as site-specific drug reservoirs. In certain conditions, they can also be used for pulmonary or topical delivery via aerosolized formulations. However, rapid clearance remains a major limitation for many applications [120]. Liposomes are employed in gene therapy and transfection, immune response enhancement, and the treatment of various diseases [121]. In advanced nanomaterial engineering, modified liposomes exhibit stimuli-responsive properties such as responsiveness to light, pH, temperature, enzymatic reaction, ultrasound, or radiation-sensitive nanoparticles; they serve as effective carriers for multimodal nanoscale trigger and effector functions [122].

#### 5.2.1. Clinical Examples

##### Doxil^®^

Pegylated liposomal doxorubicin (Doxil^®^ or Caelyx^®^) uses polyethene glycol polymers to limit reticuloendothelial uptake, resulting in increased circulation, lower distribution volume, and better tumor uptake. Preclinical studies revealed one- or two-phase plasma concentration–time profiles for pegylated liposomal doxorubicin, with an elimination half-life of 20–30 h and a volume of distribution comparable to blood volume. Due to its aqueous nature, doxorubicin is located in the inner core of the nanoparticle. The surrounding lipid bilayer acts as a protective barrier, preventing the early degradation of the medication, while the PEG coating effectively camouflages the particle from the immune system. This nanoparticle property not only prolongs the drug’s circulation in the bloodstream, but also minimizes its uptake by the mononuclear phagocyte system [123]. The AUC is enhanced 60-fold, with preferential accumulation in implanted tumors and xenografts, resulting in higher tumor drug concentrations than free doxorubicin. Clinical trials with pegylated liposomal doxorubicin in humans, including ARKS and different carcinomas, have revealed a pharmacokinetic profile with much longer circulation and reduced clearance and volume of distribution, which improves the dosage scheduling potential [124].

##### Vyxeos™ and Onivyde™

Vyxeos (CPX-351) was developed by Jazz Pharmaceuticals, Palo Alto, CA, USA. It is the first FDA-approved dual-drug containing liposomal nanomedicine that delivers cytarabine and daunorubicin in a 5:1 molar ratio. In a Phase III clinical trial in AML patients, this drug showed better potential and a higher survival rate than the conventional treatment with free cytarabine and daunorubicin [125].

Onivyde^®^ (MM-398 or PEP02), an FDA approved drug developed by Merrimack Pharmaceuticals Inc. (Cambridge, MA, USA), is made up of irinotecan containing a liposomal drug used in solid tumors and also metastatic pancreatic cancer [126].

##### Marqibo^®^

Vincristine sulfate liposome injection (VSLI; Marqibo^®^), developed by Talon Therapeutics in the USA, is a vincristine sulfate containing sphingomyelin and a cholesterol-based nanomedicine. It is mostly used in the treatment of hematologic malignancies and solid tumors such as leukemia, Hodgkin’s disease, and non-Hodgkin lymphoma (NHL).

This liposomal drug was specially engineered to address the dosing restrictions and pharmacokinetic challenges associated with traditional nonliposomal VCR. This innovative delivery method permits the administration of the higher maximum tolerable dose with accurate delivery in targeted tissues, prolongs the drug circulation time, and slows the release of the drug in the tumor interstitium [127].

### 5.3. Dendrimers

Dendrimers are highly branched, monodisperse nanostructures composed of successive layers of branching layers that resemble the concentric layer of an onion [128]. These nanostructures expand outward from a central core in a stepwise manner, leading to a controlled increase in size comparable to that of many globular proteins found in vivo. The term “generations” refers to these branching layers, which resemble a tree-like structure. Each dendrimer has an exact number of functional groups on its outer generation that can serve as a monodispersed platform to design advantageous interactions between nanoparticles and drugs and between nanoparticles and tissues. These properties have garnered substantial attention in the medical field as nanocarriers for conventional tiny medicines, DNA/RNA, proteins, and, in certain circumstances, naturally active nano-size pharmaceuticals [129]. Dendrimer-based drugs and imaging and diagnostic agents present promising options for various nanomedicine applications [130].

The PAMAM dendrimers are the most extensively used dendrimer for clinical applications [19,131]. The cationic PAMAM dendrimer interacts with a negatively charged molecule, showing a toxic effect. To reduce this effect, Jia et al. explored the role of phosphocholine and zwitterionic lipids present on the outer surface of cell membranes and found specific lipid-modified dendrimer variants that reduce adverse effects [132]. Compared to native PAMAM dendrimers, those functionalized with phosphorylcholine groups exhibit lower cytotoxicity [133]. Clinical investigations on dendrimers have been conducted. Their size makes them highly distinct from polymeric systems, micelles, and liposomes, which causes them to behave differently after systemic administration [134]. In terms of mechanical stability, dendrimers that form a single covalent bond structure surpass liposomes. They also provide an internal hydrophobic cavity for payload encapsulation and functional switching. However, compared to micelles and lipid/polymer vesicles, the payload-to-carrier weight ratio is smaller, limiting the integration of space-consuming smart sensors or nano-size switches [135]. Despite their potential, a lack of clinical expertise in dendrimer-based nanomedicine presents challenges for drug development, regulatory approval, and clinical translation, all of which influence industrial adoption and commercialization [134].

Poly(lysine) dendrimers with sulfonated naphthyl effectively inhibit herpes, HIV, and other STDs by blocking virus absorption and replication. In contrast, PPI dendrimers exhibit antibacterial properties against Gram-positive and Gram-negative bacteria [136]. Despite extensive research on dendrimers, the antibacterial effects of anionic phosphorus dendrimers remain unexplored. However, studies show that polycationic and polyanionic dendrimers enhance the activity of levofloxacin (LVFX) against *E. coli*, *P. hauseri*, and *S. aureus*, enabling reduced antibiotic doses. In drug delivery applications, Zeng et al. developed pH- and glucose-sensitive dendrimers that self-assemble into micelles, encapsulating insulin and releasing it in response to glucose changes, effectively stabilizing blood sugar levels [137]. Similarly, Bae et al. demonstrated that PAMAM-FHR dendrimers function as effective gene vectors for glioma therapy by delivering apoptin [138], while Seixas et al. improved chlorambucil’s solubility using PAMAM-NH2 dendrimers, selectively targeting prostate cancer cells without affecting colon cancer cells or fibroblasts [139]. In another study, Thuy et al. designed PAMAM G2-HRChol dendrimers incorporating histidine–arginine and cholesterol, which improved endosomal escape and gene transfection [140]. PAMAM G2-HRChol 6% exhibited high transfection efficacy with lower cytotoxicity, making it a promising gene carrier for HeLa cells. Additionally, dendrimer-cross-linked collagen gels promoted human corneal epithelial cell growth and adhesion without toxicity, unlike glutaraldehyde-cross-linked collagen, highlighting their potential as scaffolds for corneal tissue formation and tissue engineering. Furthermore, synthetic dendrimer peptides have shown promise in vaccine development. Canas-Arranz et al. demonstrated that B2T-3A and B2T-3D peptides enhance immunogenicity, producing neutralizing antibodies and IFN-producing cells in pigs, suggesting their potential for improved viral protection. These advancements underscore the versatility of dendrimers in antimicrobial therapy, drug delivery, gene therapy, tissue engineering, and vaccine applications [141].

### 5.4. Nanogels

Nanoscale hydrogels are polymeric materials composed of interlinked polymeric chains, forming a hydrophilic network that can retain and absorb large quantities of water [142]. Hydrogels closely mimic living tissue due to their high water content, soft consistency, and porosity. These versatile materials can be fabricated in various forms, for example, microparticles (MPs), slabs, coatings, nanoparticles, and films, and used in different applications. In the biomedical and clinical fields, nano hydrogels are widely used for tissue engineering, drug delivery, biomolecule separation, diagnostics, and cell immobilization [143]. They can also serve as barriers to drug accumulation, holding substantial amounts of biological fluids and swelling in the process. The ability of hydrogels to stretch and swell gives them a soft tissue-like texture, making them highly biocompatible. This unique combination of properties—water retention, flexibility, and biocompatibility—enables hydrogels to play an important role in modern medical and clinical applications [144].

One of the primary applications of hydrogels is in the production of soft contact lenses. Unlike rigid glass lenses, hydrogel-based lenses enable gas diffusion and maintain moisture on the surface of the retina, enhancing comfort and reducing dryness [145]. Clinical trials are ongoing with new hydrogel lenses for different results to improve the wear duration and added pigments and optimize the geometry of the lenses. Moreover, hyaluronic acid-containing hydrogel is used in facial correction. Juvéderm^®^ is the leading product in the market; it uses the correction of age-related volume loss and lip augmentation, and it is sensible enough to serve facial wrinkles [146].

Nano hydrogels are also utilized in biosensing applications. For instance, ionic poly(N-isopropyl acrylamide–co-methacrylic acid) (PNM) hydrogels serve as protein receptors due to their significant refractive index change upon protein binding. These hydrogels are synthesized on the surface of silica gold nanoshells (AuNSs) to create a composite material (AuNS@PNM). This combined material is employed to detect levels of two proteins, lysozyme and lactoferrin, which serve as markers for chronic dry eye. Given that lactoferrin and lysozyme have high isoelectric points, indicating their positive charge, they are attracted to the negatively charged PNM hydrogels. When these proteins attach to the hydrogels, AuNS@PNM exhibits a noticeable, concentration-dependent red shift in the Localized Surface Plasmon Resonance (LSPR) wavelength. This shift enables the identification of clinically significant changes in protein levels in human tears [147].

The physicochemical properties of nanoscale hydrogel networks, such as membrane disruption, cytocompatibility, and critical phase transition pH, can be finely tuned by adjusting the polymer composition. These tailored properties are crucial for designing intracellular drug delivery vehicles. By understanding how these vehicles internalize and function within cells, we can optimize their physicochemical characteristics to achieve more effective treatments [148].

The bifunctional disulfide cross-linker bis(2-methacryloyloxyethyl) disulfide (SSXL) was synthesized by Liechty, W.B. et al., and then pH-responsive hydrogels (PDESSB30 nanogels) were developed using DEAEMA along with the co-monomers TBMA or TBAEMA [149]. The disulfide cross-linker was designed to facilitate the degradation of pH-responsive nanogels under reductive conditions. Synthesized nanogels demonstrated the ability to efficiently deliver functional siRNA to Caco-2 cells, achieving gene silencing efficiencies of 47% and 83%, respectively, suggesting these nanogels as promising candidates for development in therapeutic siRNA delivery systems.

### 5.5. Carbon-Based Nanomaterials

Carbon-based nanomaterials including nanodiamonds, carbon nanofibers, graphene, carbon quantum dots, and carbon nanotubes, have unique qualities that make them promising for use in clinical applications [150]. These applications include the treatment of cancers that are resistant to chemotherapy, improving magnetic resonance imaging (MRI), tissue regeneration, stem cell banking, and more [151]. Furthermore, methods for enhancing the administration of drugs and imaging through carbon nanomaterials have been examined. These methods include creating endothelial leakiness and using artificial intelligence to design the nanoparticle-based drug combination delivery system [151].

#### 5.5.1. Nanodiamonds (NDs)

Nanodiamonds (NDs) are a class of carbon-based nanomaterials with unique properties that make them applicable for clinical applications [152]. NDs can be synthesized using various methods including detonation, laser ablation, chemical vapor deposition (CVD), and high-pressure and high-temperature (HPHT) techniques. Among these, detonation synthesis is the most commonly used method for the synthesis of nanoscale fluorescent NDs [153]. The fluorescence of NDs makes them applicable to several clinical applications, for example, bioimaging, diagnostic applications, and drug delivery [154,155,156]. The most significant application of nanodiamonds NDs lies in their ability to enhance the delivery of chemotherapeutic agents, particularly in chemo-resistant cancers, where drug resistance mechanisms hinder therapeutic efficacy, especially in targeting cancer stem cells (CSCs) [157]. 

NDs possess a high surface area, which enable the efficient absorption and targeted release of several anticancer drugs, for example, tetracyclines [158], 4-hydroxytamoxifen [159], and paclitaxel [160]. When encapsulated on the surface of the NDs and internalized by cells, these drug-loaded NDs help to sustain intracellular drug concentrations. This approach has been used to overcome cancer resistance to conventional chemotherapy [161]. Combination chemotherapy employing a mixture of drugs (cocktail) is the most effective treatment for tumors that are mutated and resistant to multiple drugs [162]. Recently, feedback system control (FSC) technology has been used to determine the best drug combination for ND–bleomycin, ND–DOX, unmodified paclitaxel, and ND–mitoxantrone [163]. FSC tested millions of formulations and 57 combinations. These were tested on the different types of breast cancer cell lines and consistently outperformed single drugs in every case.

Beyond chemotherapy, NDs have also gained significant attention in gene delivery applications. Gene therapy involves the transfer of genetic material to replace defective genes or enhance cellular functions [164]. NDs have been widely investigated for their applications in gene delivery, leveraging interactions between polymeric agents on their surface and negatively charged nucleic acids [165,166,167]. For example, the ND–PEI vector efficiently delivers pEGFPLuc plasmids, encoding green fluorescent protein (GFP) and luciferase into the cytoplasm [168].

#### 5.5.2. Quantum Dots (QDs)

Quantum dots (QDs) are semiconductor nanoparticles with unique shapes, tuneable sizes, and optoelectronic properties [169]. These characteristics have made QDs highly attractive in biomedical engineering for applications such as real-time bioimaging, single-molecule probes, drug delivery, intracellular tracking, in vivo imaging, and diagnostics [170]. The optical properties of QDs depend on factors such as composition, size, quantum yield, multiplexing capacity, surface area-to-volume ratio, and resistance to photobleaching. Traditional organic dyes are highly susceptible to photobleaching, and quantum yield can be less than 15% in biological environments, while QDs offer enhanced fluorescence stability and efficiency [171]. Moreover, QDs can emit fluorescence across a broad spectrum from near-ultraviolet (UV) to infrared (IR) wavelengths. QDs emitting in the near-infrared (NIR) range have significantly enhanced the potential of biomedical fluorescence imaging due to their reduced tissue absorption and comparatively low autofluorescence [171]. The primary challenge with using QDs in biomedical and theragnostic applications is their insolubility in water. To address this, many researchers aim to encapsulate QDs with hydrophilic, soluble materials such as hydrogels or polymeric matrices to improve their aqueous stability. Additionally, surface modifications, such as ligand conjugation or functionalization with hydrophilic groups, have been applied to enhance their solubility in water and biocompatibility [172]. The selection of an appropriate coating material is crucial for optimizing QDs’ performance based on the intended biomedical application and environmental conditions.

QDs can be conjugated with biomolecules such as proteins, antibodies, oligonucleotides, small-molecule ligands, and biotin-functionalized compounds to enable targeted binding, cellular uptake, and intracellular imaging [173]. These conjugated QDs facilitate precise drug delivery by enabling the controlled import and export of the therapeutics in both in vivo and in vitro systems. The conjugation efficiency depends on the surface chemistry of QDs and the functional group available for attachment, which can involve covalent or non-covalent bonding [174]. Moreover, significant work has been carried out to improve QDs’ bioconjugation strategies, focusing on achieving site-specific binding while maintaining a high quantum yield.

The CRISPR/Cas9 system is a highly effective method that enables scientists to edit the DNA of individual cells or entire organisms with precision and control [175]. In the plasmid-based CRISPR/Cas9 system, to edit genes effectively, the sgRNA and CRISPR must travel through the cell’s cytoplasm and reach the nucleus to work. Delivering CRISPR/Cas9 plasmids straight into the nucleus can make gene editing more efficient. However, getting them to the nucleus is challenging because the cell has many obstacles [176].

QDs are increasingly being used in biological applications, particularly for in vivo imaging. However, upon intravenous administration, these nano-size colloidal particles face multiple biological barriers at both the organ and cellular levels, which can limit their accumulation at target sites and reduce their efficacy [177,178]. Addressing these biological barriers is crucial for enhancing the efficacy of QDs in targeted biomedical applications.

#### 5.5.3. Carbon Nanotubes (CNTs)

Carbon nanotubes (CNTs) have garnered significant attention as a promising nanomedicine due to their distinctive and exceptional mechanical, electrical, and physicochemical properties [179]. Over the past decade, CNTs have been extensively used for applications in cancer treatment, drug delivery, bioimaging, and combination therapies [180,181]. First discovered by Sumio Iijima in 1991, CNTs are typically synthesized using methods such as arc discharge, CVD, and laser ablation [182]. The surface modification and functionalization of CNTs have been used to enhance the biocompatibility of CNTs by reducing toxicity, reducing immunogenicity, and increasing the drug loading capacity [183].

Several techniques have been explored for applying CNTs in biomedical applications. These include their use as template, immobilizing sensing agents such as antibodies and modifying their optical or electronic properties in response to specific stimuli (e.g., cancer-related proteins). Liu et al. demonstrated the application of CNTs as nanocarriers for the treatment of pancreatic and liver cancer [184]. Common biomarkers for liver cancer detection include the α-fetoprotein variant (AFP-l3), α-fetoprotein (AFP), and aberrant plasminogen (APT). Li et al. developed gold-coated CNTs that bind with antibodies labeled with a redox probe to serve as markers. The principle involves the biomarker binding to the antibody-immobilized CNTs to generate a signal. Dogu et al. reported that hepatocellular carcinoma (HCC) cells exhibit unique binding properties, whilst highly differentiated HUH7 cells demonstrate a stronger binding affinity than poorly differentiated SNU182 cells [185]. Using these characteristics, a functionalized CNT-based imaging platform was developed to assess the differentiation and invasiveness of HCC cells.

Ovarian cancer is the second most common gynecological disease worldwide, with approximately 184,000 deaths annually. Kim, M. et al. developed nanosensor arrays for the early detection of this critical disease. Their innovative device encapsulates single-walled carbon nanotubes (SWVNTs) functionalized with ssDNA, offering a highly sensitive platform for ovarian cancer diagnosis [186]. They demonstrated that high-grade serous ovarian carcinoma can be detected in serum samples from symptomatic individuals with 87% sensitivity and 98% specificity using a “disease fingerprint” derived through machine learning analysis of near-infrared fluorescence spectra emitted by an array of carbon nanotubes functionalized with quantum defects. Yang, T. et al. developed oxidized multi-walled CNTs with a large diameter to encapsulate the anticancer drug cisplatin on the inner surface. The outer surface was coated with doxorubicin (DOX), folic acid, and polyethylene glycol (PEG) to prevent the early release of cisplatin [187]. The ZnO–CNT urinary catheter effectively inhibits biofilm formation by *E. coli* and *P. aeruginosa*, reducing biofilm growth by over 50% after 120 h. Its strong antimicrobial activity suggests a cost-effective alternative to metal-coated catheters for CAUTI prevention [188]. In addition, they developed an AuNPs@SWCNTs metasurface with asymmetric resonant rings, enhancing dipole coupling and electromagnetic field strength for THz sensing. Integrated with a microfluidic channel, it achieved ultra-sensitive SAA detection (0.1 fM) with high frequency (41 GHz/fM) and amplitude (0.20/fM) sensitivity, advancing carbon-based THz biosensors [189].

Despite their promising applications, concerns remain regarding the nanotoxicology and environmental impact of CNTs, particularly due to their non-biodegradable nature. While broad FDA approval is still lacking, CNTs have been extensively studied for decades, with numerous in vivo and in vitro investigations exploring their biomedical potential [165].

### 5.6. Metallic Nanoparticles

The development of tailored nanoparticles has advanced nanotechnology, particularly in biomedical applications. Because of their high stability and nanoscale dimensions, metallic nanoparticles have been thoroughly investigated compared to other nanomaterials [190]. By optimizing specific particle parameters, including size, shape, aspect ratio, functionalization, and synthesis techniques, their intrinsic properties like their optical and electrical properties, surface plasmon resonance, and other physicochemical characteristics can be modified [190]. These tunable features enable diverse applications, including biosensing, drug delivery, photodynamic therapy, imaging, and multimodal therapeutic strategies. The unique properties of metallic NPs have been particularly explored in cancer treatment [191,192].

Metallic nanoparticles are designed to detect and treat cancer by binding to cancer cells, improving imaging and enabling targeted treatment with fewer side effects [193]. Gold nanoparticles (AuNPs) aid in gastrointestinal (GI) cancer detection by enhancing CT imaging and delivering drugs directly to tumors. Iron oxide nanoparticles (IONPs) target HER2-positive breast cancer cells, improving MRI and using hyperthermia to destroy cancer cells. Silver nanoparticles (AgNPs) detect lung cancer by binding to EGFR receptors, enhancing imaging through surface-enhanced Raman scattering (SERS) and delivering drugs to tumors. Platinum nanoparticles (PtNPs) help to detect ovarian cancer by targeting cancer cells with folic acid, enhancing CT imaging and enabling photothermal therapy. Zinc oxide nanoparticles (ZnONPs) improve prostate cancer detection by binding to PSMA antibodies, enhancing fluorescence imaging and allowing for targeted drug delivery. Copper oxide nanoparticles (CuONPs) assist in colorectal cancer detection by using aptamers to recognize cancer cells, improving imaging through photoacoustic tomography (PAT), and supporting photodynamic therapy. These nanoparticles offer significant cancer diagnosis and treatment advancements, making therapies more precise and effective [194,195,196].

Gold nanoparticles (AuNPs) deliver drugs like doxorubicin, releasing them in response to pH changes in tumors. Cell viability is measured using MTT or Trypan Blue assays. Iron oxide nanoparticles (IONPs) release drugs through magnetic field-induced hyperthermia, with cell viability assessed using the Alamar Blue assay. Silver nanoparticles (AgNPs) deliver drugs via light-triggered release (photothermal therapy), and their effect is monitored using flow cytometry. Platinum nanoparticles (PtNPs) release cisplatin in acidic cancer environments, with cell viability measured using the CellTiter-Glo assay, which detects ATP levels [197].

#### 5.6.1. Clinical Examples

##### NanoTherm

Nanotherm is a magnetic nanoparticle, developed by MagForce Nanotechnologies AG in Germany. This drug is made of superparamagnetic iron oxide nanoparticles (SPIONs) coated with amino silane and is used for cancer thermal therapy [198,199]. The treatment uses localized heating (41–46 °C) to make cancer cells more sensitive to therapy or applies higher heat (>46 °C) to directly destroy them and the surrounding tissues [200]. We have summerized some of clinically approved nano-based approaches (Table 1) and those currently in clinical trials (Table 2) bellow:

## 6. Limitations of Nanomaterials

**Figure 4:**
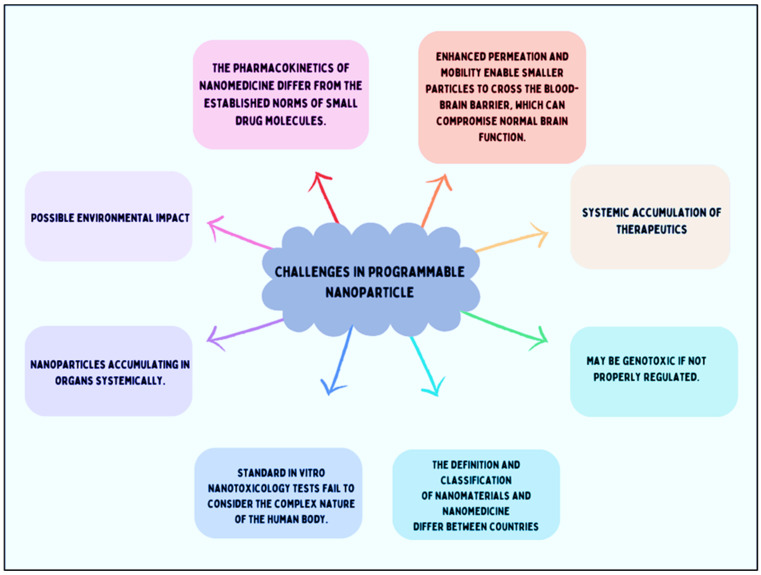
Challenges in programable nanoparticles.

Nanomedicine has evolved over the past several decades from biologically inert materials to intelligent systems designed to enhance in vivo functionality [201]. Many nanomaterials directly interact with genetic material, and these nanoparticles’ interaction with biomolecules helps in normal cell division and gene function [202]. However, these interactions may also lead to mutagenicity and toxicity [203]. Currently, many interactions between nanoparticles and biological systems remain unclear. As a result, deciphering, characterizing, or making inferences about the physicochemical and toxicological properties of nanomedicines remains challenging. This is an area where progress is likely to be gradual in the absence of standard regulatory guidelines. It is crucial to understand that there is no “one size fits all” solution, because the distinct characteristics seen at the nanoscale rely heavily on the kind of nanoparticle, its surface characteristics, how it is administered, and most importantly, its variety of morphologies. Because of this variability, the regulatory procedure is very intricate. Cellular and nanotoxicity responses are another challenge faced [204] (Figure 4).

There are several ideas for overcoming cytotoxicity. Traditional toxicity testing on large animals, which was previously used for small drug molecules, has been deemed unethical, excessively expensive, and impractical for evaluating nanotoxicity [205]. In vitro toxicity techniques are employed to assess nanoparticles. They offer cost-effective and time-efficient managed experimental conditions compared to animal testing. However, various assays are utilized to bypass the complexities of the human body [206], which employs compensatory mechanisms and pathological responses to handle toxins, along with intricate metabolic processes. Additionally, growing evidence indicates that traditional in vitro tests for small compounds are unsuitable for nanomaterials [207]. Nanomaterials interact with the reagents used in in vitro assays. Their characteristics—such as optical properties, high absorption, acidity or alkalinity, catalytic activity, dissolution, and magnetic properties—result in interactions with the reagents in these tests [208]. Consequently, new tests are necessary to evaluate the toxicity of nanomaterials and nanomedicines before the appropriate regulatory guidelines can be established. This need is significantly hindering progress in the field [209]. When designing nanoparticles for clinical applications, the drug delivery process and mechanism require preclinical trial data for approval, including information on advanced effects.

In 2009, Owen and Raynard highlighted that a single-size standard is not suitable for all types of nanoparticles, due to differences in clinical requirements, physiology [210], and application routes. However, this warning has been largely ignored by the current regulatory authorities. The complexity of the structure, size, form, and clinical application of programmable nanoparticles poses significant challenges to the regulatory system in classifying and characterizing nanoparticles. For example, dynamic light scattering (DLS) is used to determine hydrodynamic size, but this technique assumes that the particles scattering light are spherical, making it inaccurate for rod-shaped materials. Additionally, other techniques used for size measurement may not accurately reflect the form of nanomaterials experienced in the human body. For instance, using a transmission electron microscope involves drying samples, which may alter their shape or size compared to their form in solution. Protein coronas commonly form when nanomaterials are injected into the bloodstream, leading to significant underestimation of their true size in a physiological context. There is no consensus in the literature on the best standards for nanometrology or characterization [211]. As a result, preclinical nanomedicine development will likely continue without stringent clinical regulatory guidance or intervention.

Nanomedicines exhibit unconventional behavior compared to small drug molecules, leading to prolonged bioavailability. This extended presence in the body could present substantial health risks if these products were to be sold over the counter. Consequently, the regulatory authorities must meticulously assess whether nanomedicines warrant strict monitoring or can be available as over-the-counter products. However, arriving at a definitive decision is difficult due to the current absence of toxicity data and information.

## 7. Conclusions and Outlook

The rapid progression of nanotechnology has significantly bolstered preclinical development, especially in the area of nanomedicine. Programmable nanomaterials have important characteristics, for example, shape, fluorescence, size, mechanical strength, surface chemistry, and surface area. These characteristics help to improve drug delivery, tissue engineering, bioimaging, nanomedicine, therapeutics, and numerous other biological applications. Nanoparticles should possess characteristics such as biocompatibility, bioavailability, biodegradability, and targeted and controlled drug release that exceed those of their larger counterparts. These properties are provided to address difficult challenges and improve therapeutics. Polymeric nanomaterials, liposomes, dendrimers, and nano hydrogels are at the forefront of the revolution. Carbon-based NPs, for example, nanodiamonds, carbon quantum dots, and carbon nanotubes offer unique electronic, magnetic, and optical properties that are highly advantageous for drug delivery, bioimaging, and cancer treatment. Nanodiamonds have shown promise in chemotherapy resistance and fascinating gene delivery, underscoring their potential in treating resistant and genetic diseases. Metallic nanoparticles have potential biological applications.

Despite these advancements, translating nanomedicine from research into clinical practice faces significant challenges. One major hurdle is the biological barriers that nanoparticles encounter, affecting their distribution, cellular uptake, and overall efficacy. The shape, size, and surface chemistry of nanoparticles must be carefully engineered to maximize their interactions with biological systems. Looking ahead, the future of programmable nanomaterials in clinical applications is promising, yet contingent on overcoming these challenges. Continued research and development are essential to optimize nanoparticle design, enhance targeting and delivery mechanisms, and mitigate potential toxicity. Collaboration between researchers, clinicians, and regulatory authorities will be crucial in establishing comprehensive guidelines and protocols for the safe and effective use of nanomedicines. In conclusion, targeted nanomaterials have promising clinical applications in the biomedical sector.

## Figures and Tables

**Table 1:** Some examples of clinically approved nanomedicines.

Name	Drug	Nanocarrier	Application	Manufacturing Company
Myocet^®^	Doxorubicin	Liposome	Breast cancer	Teva
Marqibo^®^	Vincristine sulfate	Liposome	Acute lymphoblastic leukemia (ALL)	Talon Therapeutics
Ambisome	Amphotericin B	Liposome	Fungal infection	Gilead Sciences
Onivyde^®^	Irinotecan	Liposome	Pancreatic cancer	Merrimack Pharmaceuticals
Doxil^®^	Doxorubicin	Liposome	Various cancers, ALL, AML, breast cancer, ovarian cancer	Johnson & Johnson
Genexol PM^®^	Paclitaxel	mPEG–PLA	Metastatic breast cancer	Samyang Corporation
Eligard^®^	Leuprolide acetate	PLGA	Prostate cancer	Tolmar
Vyxeos^®^	Cytarabine and daunorubicin	Liposome	AML	Jazz Pharmaceuticals
Abraxane^®^	Paclitaxel	Albumin	Various cancers, breast cancer	Abraxis BioScience

**Table 2:** Nanomedicines in clinical trials in Phases II and III.

Name	Drug	Nanocarrier	Application in	Manufacture Company
Genexol-PM^®^	Paclitaxel	Polymeric micelles	Breast, lung, and ovarian cancer	Samyang
NK-105^®^	Paclitaxel	Micelle: PEG– polyaspartate	Metastatic breast cancer	Nippon Kayaku Co.
NanoTherm	Aminosilane- coated SPIONs	Metallic nanoparticle	GBM and prostate cancer	MagForce Nanotechnologies
ThermoDox	Doxorubicin	Thermosensitive liposome	Hepatocellular carcinoma	Celsion
Lipoplatin^®^	Cisplatin	Liposome	Breast, pancreatic, urinary bladder, and gastrointestinal cancer	Regulon Inc.
